# High prevalence of schistosomiasis in Mbita and its adjacent islands of Lake Victoria, western Kenya

**DOI:** 10.1186/1756-3305-5-278

**Published:** 2012-12-03

**Authors:** Maurice R Odiere, Fredrick O Rawago, Maurice Ombok, William Evan Secor, Diana MS Karanja, Pauline NM Mwinzi, Patrick J Lammie, Kimberly Won

**Affiliations:** 1 Neglected Tropical Diseases Branch, Centre for Global Health Research, Kenya Medical Research Institute, P. O. Box 1578–40100, Kisumu, Kenya; 2 Centers for Disease Control and Prevention, Division of Parasitic Diseases and Malaria, 1600 Clifton Rd, N.E.; Mailstop-D65, Atlanta, GA, 30329-4018, USA

**Keywords:** Geographical distribution, Island, Schistosomiasis, Soil-transmitted helminths, Western Kenya

## Abstract

**Background:**

Intestinal schistosomiasis continues to be a significant cause of morbidity among communities located around Lake Victoria and on its islands. Although epidemiological surveys have been conducted in other areas bordering the lake in western Kenya, Mbita district and its adjacent islands have never been surveyed, largely due to logistical challenges in accessing these areas. Consequently, there is a paucity of data on prevalence of schistosomiasis and soil-transmitted helminth (STH) infections that are endemic in this region.

**Methods:**

This cross-sectional study determined the prevalence, intensity of infection and geographical distribution of schistosome and STH infections among 4,065 children aged 5–19 years in 84 primary schools in Mbita and nearby islands of Lake Victoria (Mfangano, Ringiti, Rusinga and Takawiri), in western Kenya. Single stool samples were collected and examined for eggs of *Schistosoma mansoni* and STHs (Hookworms, *Ascaris lumbricoides and Trichuris trichiura*) using the Kato-Katz technique. Primary schools were mapped using geographical information system data on PDAs and prevalence maps generated using ArcView GIS software.

**Results:**

Overall, 65.6% (95% CI = 64.2-67.1%) of children were infected with one or more helminth species; 12.4% (95% CI = 11.4-13.4%) of children were infected with one or more STH species. Mean school prevalence of *S. mansoni* infection was 60.5% (95% CI = 59.0-62.0%), hookworms 8.4% (95% CI = 7.6-9.3%), *A. lumbricoides* 3.3% (95% CI = 2.7-3.8%), and *T. trichiura* 1.6% (95% CI = 1.2-2.0%). Interestingly, the mean *S. mansoni* prevalence was 2-fold higher on the islands (82%) compared to the mainland (41%) (z = 5.8755, *P* < 0.0001). Similarly, intensity of infection was 54% higher on the islands (217.2 ± 99.3) compared to the mainland (141.3 ± 123.7) (z = 3.9374, *P* < 0.0001). Schools in closest proximity to Lake Victoria had the highest *S. mansoni* prevalence while prevalence of STHs was more homogenously distributed.

**Conclusions:**

The very high prevalence of schistosomiasis in Mbita and the 4 islands is quite alarming, and indicates an urgent and critical need for control interventions. Findings from this survey indicate the need to implement treatment in remote areas not previously covered by mass drug administration programs.

## Background

Communities around the shores of Lake Victoria in western Kenya are populated with individuals who suffer significant morbidities associated with *Schistosoma mansoni* infection [1,2], this despite the availability of effective and safe drugs, and the fact that mass treatment of school-aged children is hailed as a cornerstone of schistosomiasis and soil-transmitted helminth (STH) control activities [3,4]. The situation is further exacerbated in remote areas and Islands on Lake Victoria that have perennially received few health interventions, due in part to logistical challenges in accessing these areas. In addition, such rural areas are usually underserved and marginalized, and their voices are not heard sufficiently to firmly establish theneed for interventions targeting schistosomiasis and STHs within the national political agenda [5].

In Kenya, the Ministry of Health’s Department of Child Health seeks to promote good health and nutrition, and it recognizes the detrimental effects of helminth infections in primary-school-aged children [6]. However, several challenges exist towards the implementation of cost-effective helminth control strategies. First, while schistosomiasis tends to be focal in distribution, there is inadequate research on the geographical distribution of both schistosome and STH infections, and a poor understanding of helminth epidemiology in rural settings. Second, although schistosomiasis among communities residing on the mainland is closely associated with proximity to the Lake Victoria in western Kenya, data on schistosome infections on the islands are remarkably lacking. The picture is further confounded by the highly itinerant population within these areas [7], thus complicating efforts to define the pattern for locally acquired versus imported infections on the islands. In addition, molecular studies around Lake Victoria have revealed higher genetic diversity of *S. mansoni*[8,9], with the potential to modify the clinical pattern and morbidity among infected individuals.

The World Health Organization (WHO) has emphasized the need to create predictive maps for expected schistosome and STH distributions. In this regard, geographical information system (GIS) data can be applied to consider the spatial patterns of human infection so as to improve efficient allocation of available transmission control interventions. This effort is best exemplified through the recently launched Global Atlas of Helminth Infections (GAHI) [10].

The objective of this cross-sectional study was to determine the prevalence, intensity of infection and geographical distribution of schistosome and STH infections among primary school children in Mbita and surrounding Islands of Lake Victoria (Mfangano, Ringiti, Rusinga and Takawiri) in western Kenya. Understanding the prevalence and distribution of infection in such endemic areas not only serves as a guide in pin-pointing high-risk populations and transmission sites which is critical in developing sound and targeted control interventions to reduce the burden of these infections, but also to provide a robust baseline to monitor program impact.

## Methods

### Study area

The study was conducted in Mbita district, which borders Lake Victoria in western Kenya, between February-March 2012. In line with the new Kenyan constitution, Mbita district is now part of Homabay County. Mbita is located at latitude (0°25’S) and longitude (34°12'E), has 3 main divisions (Mbita, Lambwe & Mfangano) and has a population estimated at 111,409 [11]. Its associated Islands include Mfangano, Remba, Ringiti, Rusinga and Takawiri. Rainfall pattern in Mbita is seasonally bimodal, with the heaviest rains falling from March through May and the shorter rainy season occurring between September and December. On the highlands the rainfall ranges between 800-1900 mm per annum; in lower areas, it is between 800-1200 mm each year. The mean minimum temperature is 15°C while the mean maximum temperature is 30°C.

Though there are many development initiatives in Mbita, poverty is still a major challenge. More than three quarters of the population survives on less than 1 USD/day, the World Bank's definition of extreme poverty [12]. The majority of the Mbita population and islanders live along the lake in beach communities as the main economic activity is fishing. In addition, the lake is used for washing clothing and dishes, bathing, washing cars, sand harvesting, and irrigating farmland. Farming is mostly subsistence-based, with major crops including sorghum, potatoes, cassava, beans and maize. In terms of healthcare infrastructure, a district hospital is located within the town, with a few other government dispensaries and private clinics.

### Study design

Based on previous evidence of an inverse association between prevalence of schistosomiais and proximity to the lake [2], and the goal to enroll schools with ≥ 25% prevalence for schistosomiasis in a long term control study, 84 public primary schools located within a radius of 5Km from the lakeshore were selected for participation in the study. All public primary schools on the 4 islands (n = 37) were selected to participate in the study. This cross-sectional survey involved a random selection of 50 students in classes 7 and 8 from a list of registered students obtained from the headteacher, using a random number generator. In schools where there were fewer than 50 students per class, all the students were included in the survey. In schools that did not include class 7 and 8, selection into the study was based on the highest 2 classes at the school. Informed consent was obtained from the parent or guardian and assent was obtained from the student prior to enrolment in the survey. Children infected with schistosomes were treated with 40 mg/kg praziquantel (PZQ) and those infected with STHs were treated with 400 mg albendazole (ALB). Prior to the current study, there was one school-based national deworming conducted in May 2009, where a single dose of ALB (400 mg) was administered. The current study was reviewed and approved by the Scientific and Ethical Review Committees (ERC) of the Kenya Medical Research Institute (KEMRI, SSC No. 2185). The Institutional Review Board of the Centers for Disease Control and Prevention also reviewed the study and chose to rely on the KEMRI ERC approval.

### Geographical distribution of infections

To determine the geographical distribution of the infection prevalence, positions of all primary schools participating in the study area were mapped using hand-held differential geographic global positioning system (GPS) units (Trimble Navigation Ltd, California, USA) with an estimated accuracy of ± 1 meter [13]. Data were downloaded with differential correction into a GPS database (GPS pathfinder office 2.8 Trimble Navigation Ltd, California, USA) and analyses performed using ArcView version 9.2 software (Environmental Systems Research Institute, Inc., Redlands, CA). Mapped school prevalence was categorized according to WHO prevalence thresholds for mass drug administration [14], with an added category denoting zero prevalence: 0%, 0.1-9.9%, 10–49.9% and 50-100%.

### Parasitological assessment

Parasitological assessment was based on one stool per child. Plastic stool containers were given to children on the morning of the day for the survey, and students were instructed to bring samples of their stool. Up to 90% of children in a given school (range = 82-100%) returned a stool sample. All stool samples were transported to The Ministry of Health’s Division of Vector-Borne Diseases (DVBD) laboratory, Homabay, where they were processed the same day. Each stool sample was analyzed in duplicate by the Kato-Katz method for eggs of *S. mansoni, A. lumbricoides*, *T. trichiura* and hookworms [15]. A template was used that when filled contained approximately 41.7 mg of feces. Eggs were counted by two independent microscopists and any discrepancy in results of the two was reconciled by comparing to results of a third independent and more experienced microscopist. Examination of slides for hookworm eggs were performed within 1 h of slide preparation. For all other helminths, slides were allowed to clear for at least 24 h and eggs were counted within one month. The concentration of eggs was expressed as eggs per gram (epg) of feces. Intensity of infection for each helminth was categorized according to the World Health Organization (WHO) proposed thresholds [14].

### Data analysis

All analyses were performed using SAS statistical software (v. 9.2; SAS Institute Inc., Cary, NC, USA) and *P* values < 0.05 were considered statistically significant. Unless otherwise indicated, values are presented as means ± SD. Data were checked for normality and log-transformed [log (x + 1)] when necessary, but only non-transformed means are reported. The Wilcoxon-Mann–Whitney test was used to compare the difference in prevalence of infection and egg density between schools on the mainland and those on islands. The effect of age and gender on whether a child was infected was analysed using logistic regression. Given the typical overdispersion of egg counts, Kruskal-Wallis analysis was used to assess the variation of egg counts by age and gender.

## Results

### Geographical distribution of infections

A total of 4,064 children (from 37 schools on the islands and 47 schools on the mainland) were included in the survey, with ages ranging from 5 to 19 years. The mean age was 12.6 and the median was 13 years.

**Figure 1:**
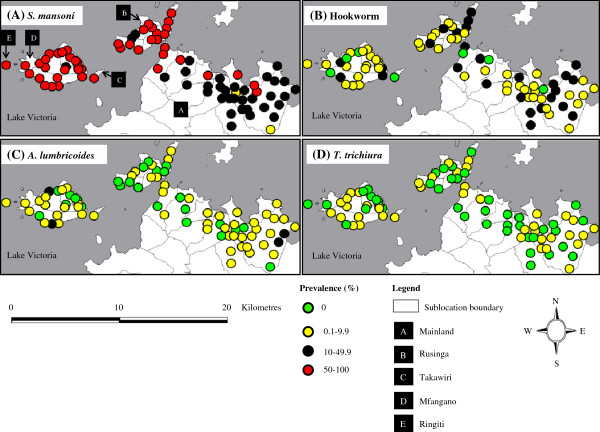
**Geographical distribution of *Schistosoma mansoni*, hookworm, *Ascaris lumbricoides *and *Trichuris trichiura *among schools in Mbita and adjacent islands, western Kenya.**

As observed in previous studies, schools in closest proximity to Lake Victoria had the highest *S. mansoni* prevalence while schools with STH infections were more homogenously distributed (Figure 1). Whereas *S. mansoni* was present in all schools, STHs were absent in several schools; hookworm in 8, *A. lumbricoides* in 25 while *T. trichiura* was absent in 42 schools (Figure 1).

### Parasitologic outcomes

The most prevalent helminth infection among the children was *S. mansoni* (60.5%) (Table 1). All but 2 schools had an *S. mansoni* prevalence ≥ 10%, warranting some form of MDA; 34 schools (40.5%) had prevalence between 10% and 50% and 48 schools (57%) had *S. mansoni* prevalence ≥ 50%. Two schools located on Rusinga island (Wanyama and Waregi) had 100% prevalence (Figure 1A). The prevalence of STH species was ≥ 10% in 50 schools (59.5%), and was ≥ 20% in 13 schools (15.5%). No school had STH prevalence > 50%. *S. mansoni* infections were mainly light and moderate, whereas STH infections were all light, except for *A. lumbricoides* (Table 1). Overall, 65.6% of the children were infected with either *S. mansoni* or one of the STHs, while only 12.4% of children were infected with one or more STH species, but not schistosomes (Table 1).

**Table 1:** **Prevalence and intensity of infection for schistosomiasis and soil-transmitted helminths among children 5–19 years old in Mbita and nearby islands, western Kenya**^**1**^

**Species infection**	**Overall prevalence, (%)**^**2**^	**Intensity threshold prevalence, (%)**			**Intensity (epg)**^**3**^
		**Light**	**Moderate**	**Heavy**	
One or more helminth	65.6 (64.2-67.1)				
*S. mansoni*	60.5 (59.0-62.0)	49.0	35.8	15.2	215.8 ± 343.5
One or more soil-transmitted helminth	12.4 (11.4-13.4)				
Hookworm	8.4 (7.6-9.3)	100	0.0	0.0	47.7 ± 87.9
*A. lumbricoides*	3.3 (2.7-3.8)	80.3	19.7	0.0	2580.5 ± 4283.2
*T. trichiura*	1.6 (1.2-2.0)	100	0.0	0.0	30.1 ± 43.9

^1^ Sample size: 5–7 years-110, 8–10 years-406, 11–13 years-2129, 14–16 years-1328, 17–19 years-29, unknown age-62.

^2^ Values in parenthesis indicate 95% confidence intervals.

^3^ Intensity of infection expressed as arithmetic mean ± SD.

**Figure 2:**
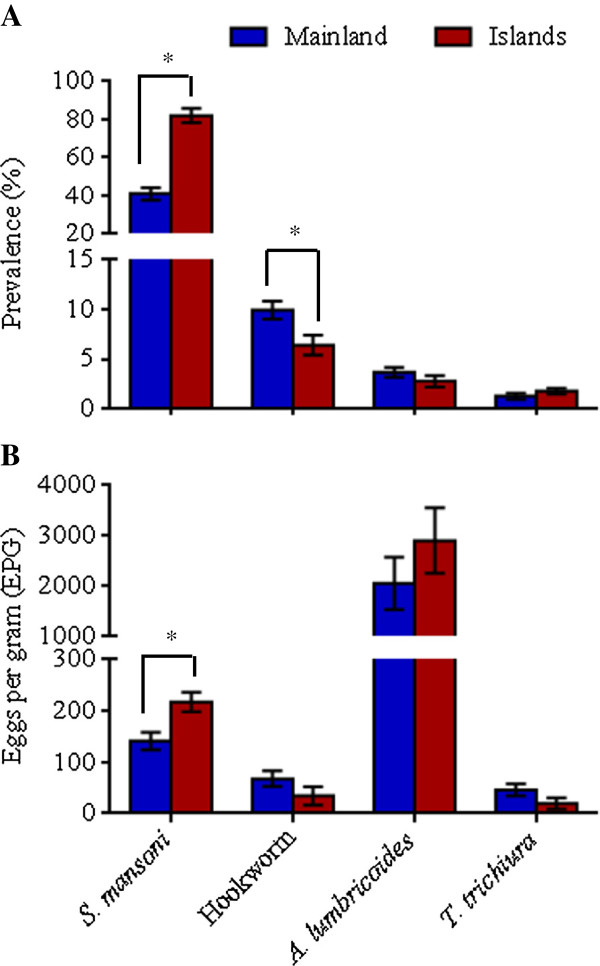
**Comparison of prevalence of infection and egg density for helminths between Mbita mainland and islands.** Values are mean ± SEM.

Mainland versus islands stratification of schools revealed an interesting picture. The mean *S. mansoni* prevalence was 2-fold higher on the islands (82%) compared to the mainland (41%) (z = 5.8755, *P* < 0.0001) (Figure 2A).Similarly, the *S. mansoni* egg density was 54% higher on the Islands (217.2 ± 99.3) compared to the mainland (141.3 ± 123.7) (z = 3.9374, *P* < 0.0001) (Figure 2B). All schools on the Islands had *S. mansoni* prevalence ≥ 10%, and 34 out of 37 (92%) had *S. mansoni* prevalence ≥ 50%. The overall STH prevalence on the islands was 10.6% compared to 13.8% on the mainland, but the only significant difference in prevalence was seen with hookworm infection (*F*_1, 82_ = 6.39, *P* = 0.0134, Figure 2A). In terms of intensities of infection, one quarter of the infected children (mainland and islands combined) harboured heavy *S. mansoni* infections, whereas infections for the STHs were light, except for *A. lumbricoides* with ~20% of the children displaying moderate infections (Table 1).

**Figure 3:**
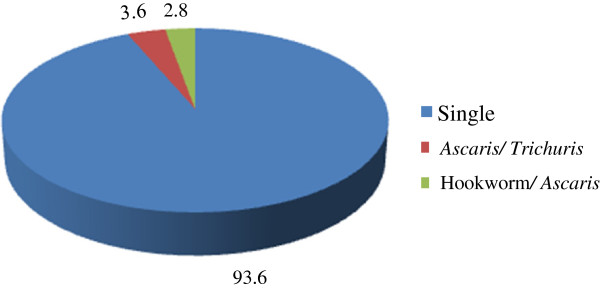
Prevalence (%) of single soil-transmitted helminths and co-infections among 503 infected primary school children in Mbita and adjacent islands, western Kenya.

Infection with multiple helminth species was also assessed. Out of the 2,666 infected children, 88.4% harboured a single helminth species, 11.1%, two species, 0.5%, three species and 0.04% were infected with all 4 species. Two hundred and ninety three (11%) children had *S. mansoni* and at least one or more STH infection. Among the *S. mansoni*-STH dual infections (280 children), *S. mansoni*-hookworm was the most common co-infection (67.9%) followed by *S. mansoni-Ascaris* (18.9%) and *S. mansoni-Trichuris* (13.2%). Among the *S. mansoni*-STH triple infections (13 children), *S. mansoni-Ascaris-Trichuris* were the most common co-infection (69.2%) followed by *S. mansoni*-hookworm-*Trichuris* (30.8%). The distribution of STH co-infections among infected children is shown in Figure 3. *Ascaris*-*Trichuris* was the most common STH only co-infection observed in children. None of the children harboured hookworm-*Trichuris* co-infection and none had all the three STHs in the absence of schistosome co-infection (Figure 3).

**Figure 4:**
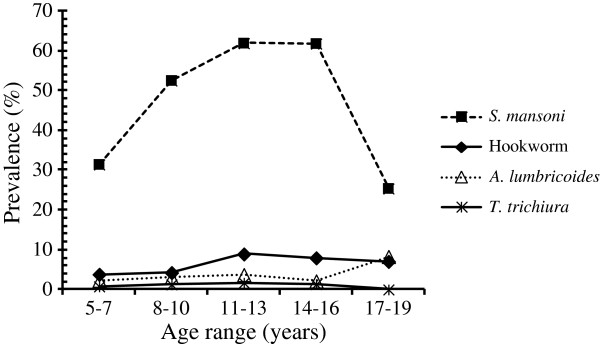
Prevalence of S. mansoni and soil-transmitted helminths in Mbita and adjacent islands by age.

We also determined the prevalence of schistosome and STH infections among the study population by age. Among all the helminths surveyed, *S. mansoni* had the highest prevalence across all ages (Figure 4). Prevalence increased from 31.4% among children aged 5–7 years to peak at 61.8% among children aged 11–16 years before declining in the 17–19 year old group(Figure 4). The typical age-dependent curve was observed for all STHs, although prevalence tended to increase for *A. lumbricoides* among the 17–19 year olds (Figure 4).

Analyses of prevalence and intensity of infection revealed no significant differences by gender (data not shown).

## Discussion

This cross-sectional survey highlights the significant burden and high endemicity of intestinal schistosomiasis in Mbita and on its islands, and outlines the distribution of infection in areas that have received very little prior attention. Close to two-thirds of the school children in the overall study were infected with one or more helminths, predominantly with *S. mansoni*. The 4 islands revealed a worse picture for *S. mansoni*, with more than 8 out of 10 children infected. Prevalence of STHs was relatively low, with about 1 in 8 of the children infected with one or more STH. Consistent with previous research [2], our data supports the inverse association between proximity to the lake and *S. mansoni* prevalence in this setting. STH infections were more or less homogenously distributed.

### Prevalence and intensity of *S. mansoni* and STH infections

The mean school prevalence of *S. mansoni* in our study (60.5%) was slightly lower than that from a study in Tanzania (64.3%) [16], but was higher compared to studies from western Kenya (16.3% and 38.8%) [2,17] and from Sesse Islands of Lake Victoria, Uganda (34.6%) [7]. Differences may be due to the focal distribution of *S. mansoni*[18], proximity selection (< 5Km) of schools to Lake Victoria, and the inclusion of a much older age range in our study (> 15 years). Prior to this study, there had been no mass drug administration to treat schistosomiasis in this area.

 In contrast, the STH prevalence for schools in our study was lower compared to other studies in western Kenya [2,19] and on Pemba Island [20]. Although the prevalence of hookworm infections observed in the current study area (8.4%) was much lower than that reported in Tanzania (38%) [16] and Western Kenya (42.5%) [2], our data supports their observations that hookworm is the predominant STH infection within the Lake basin. Two observations may explain the relatively low STH prevalence in our study, First, the direct smear microscopic analysis of single stool samples may have missed light infections because of poor sensitivity and day-to-day fluctuation in egg excretion [21]; thus, 12.4% is a minimum estimate of prevalence. Future surveys may be enhanced by examining stool samples collected for at least two consecutive days or use of concentration techniques. Second, the low STH prevalence observed in our study may be attributable to the Kenya National deworming exercise conducted in 2009, in which most of the schools in our study participated.

Infection intensities were predominantly light for *S. mansoni* and *A. lumbricoides* and were all light for hookworm and *T. trichiura*, supporting previous observations that most individuals in an endemic community excrete low numbers of eggs [22]. Our study was also consistent with age-prevalence curves from other studies that peak in early adolescence for both *S. mansoni* and STH prevalence [23].

### Helminth co-infections

An overlap of helminth species was also observed in our study. About 11% of children harboured both *S. mansoni* and at least one STH infection, which was lower than what was observed elsewhere [2,24]. Multiple STH infections in the current study was consistent with our previous findings in an urban setting close to the Lake [19], but were less common than in other studies around the lake in East Africa. In Busia, Kenya, 26% of children were infected with all 3 STHs and 31.1% with 2 [24]. On Pemba Island of Lake Victoria, Tanzania, 67% of children were infected with all 3 STHs and 28% with 2 [20]. Such differences might arise from differences in study subjects, socio-demographic conditions and socio-economic characteristics of the areas, or might be associated with differences in conditions that favour multiple parasitic species survival and transmission, including poverty, environmental contamination, water bodies and lack of effective preventive measures [25]. The highest STH co-infection prevalence observed for *Ascaris* and *Trichuris* in the current study is consistent with our previous findings [19] and supports findings by Booth and Bundy [26] that these two species have a closely related distribution. Multiple helminth infections have an impact on morbidity and overall health. Children with multiple helminth infections, especially those with heavy infection intensities, tend to experience more severe cognitive outcomes and other health problems, such as malnutrition, than children with only one helminth infection [27].

### Implications for mass drug administration programs

The prevalence and distribution of schistosomiasis and STH infections in our study has several implications for mass treatment programs. WHO recommends mass drug administration with PZQ (for schistosomes) and ALB or mebendazole (MBD) for STH wherever the prevalence of infection exceeds 10% and 20%, respectively [28]. Following this recommendation, 82 (96%) of the schools in our study require mass treatment for schistosomiasis. Of the 4,064 school children surveyed, 3,971 (97.7%) would benefit from mass treatment. This would include 2,449 (99.6%) of 2,458 children infected with intestinal schistosomiasis. Forty eight schools (*S. mansoni* prevalence > 50%, classified as high risk) and 34 schools (*S. mansoni* prevalence ≥ 20% but < 50%, classified as moderate risk) would require treatment once a year and once every 2 years, respectively [28].

In our study, 13 (15.5%) of the schools (STH prevalence ≥ 20% but < 50%, classified as low risk) would require mass treatment for STH once each year [28]. Of the 4,064 school children surveyed, 618 (15.2%) would benefit from mass treatment. This would include 147 (29.2%) of 504 children infected with STH. Based on our findings, 13 schools would require co-administration of PZQ and ALB or MLB, and 69 schools would require PZQ.

## Conclusions

Our findings show that Mbita and the nearby islands in Lake Victoria are high endemic areas for schistosomiasis, and call for urgent control interventions, key among them chemotherapy. Findings from this survey should be utilized in advocating for extending treatment benefits to areas not previously covered by mass drug administration programs. Health education, strengthening of basic infrastructure, adequate clean water and sanitation, and community involvement are advocated for to complement chemotherapy.

## Abbreviations

CDC: Centers for Disease Control and Prevention; DVBNTD: Division of Vector-Borne and Neglected Tropical Diseases; GIS: Geographical information system; GPS: Global positioning system; WHO: World Health Organization.

## Competing interests

The authors declare that they have no competing interests.

## Author’s contributions

The study was designed by MRO, PNMM, PJL and KW. FRO co-ordinated the fieldwork and labwork. MO and MRO analyzed the GIS data and generated the maps. All data was compiled and analyzed by MRO. PNMM, WES, DMS and PJL provided scientific guidance in data collection, planning and implementation of day-to-day field and laboratory activities. The manuscript was prepared by MRO, all authors actively contributed to the interpretation of the findings. All authors read and approved the final manuscript.
